# Use of Natural Compounds in the Management of Diabetic Peripheral Neuropathy

**DOI:** 10.3390/molecules19032877

**Published:** 2014-03-05

**Authors:** Galuppo Maria, Giacoppo Sabrina, Bramanti Placido, Emanuela Mazzon

**Affiliations:** IRCCS Centro Neurolesi “Bonino-Pulejo”, Via Provinciale Palermo, S.S.113, Contrada Casazza, Messina 98124, Italy

**Keywords:** diabetes, diabetic peripheral neuropathy, neuropathic pain, complementary and alternative medicine, phenolic compounds, cannabinoids, alkaloids

## Abstract

Nephropathy, retinopathy cardiomyopathy and peripheral neuropathy are all recognized as important complications in about 50% of diabetes mellitus (DM) patients, mostly related to a poor glycemic control or to an improper management of this pathology. In any case, amongst others, diabetic peripheral neuropathy (DPN) seems the leading and most painful complication usually affecting many DM patients. For this reason, this work was conceived to review the large variety of strategies adopted for management of DPN, starting from the most conventional therapies to arrive at alternative approaches. From this perspective, both the most popular pharmacological treatments used to respond to the poorly effect of common analgesics—non-steroidal anti-inflammatory drugs (NSAIDS) and opioids—understood as gabapentin *vs.* pregabalin clinical use, and the guidelines provided by Oriental Medicine as well as by a long list of natural compounds that many authors identify as possible therapeutic or alternative agents to replace or to combine with the existing therapies will be included. Moreover, in the effort to provide the widest panel of remedies, the most antique techniques of acupuncture and electrostimulation will be considered as alternative, which are useful approaches to take into account in any non-pharmacological strategy for DPN management.

## 1. Introduction

Today, among metabolic disorders, diabetes can be considered the most common, increasing, serious and costly public health problem in the World [1,2], both for the disease *per se* and for its severe secondary complications. Among them, microangiopathies leading to nephropathies, optic nerve damage associated to retinopathies and diabetic peripheral neuropathy (DPN) associated to pain [2], tingling, or numbness, loss of feeling in the hands, arms, feet and legs can occur primarily due to uncontrolled diabetes levels [3]. Moreover, in the most troubling cases this can result in more severe macroangiopathies and outcomes such as stroke and ictus [4]. 

With particular regard to the secondary neurologic damage caused by the disease, the efforts of these authors will be aimed at addressing the increasingly serious problem of DPN. Patients with symptomatic DPN usually can display spontaneous positive (paresthesia described as prickling, tingling, “pins and needles” burning, crawling, itching, abnormal sensation to temperature, pain), or negative (numbness, injury insensitivity) sensory symptoms in the toes. With time, such sensations may advance up the foot and leg and involve the fingers and hands. Neuropathic pain is a prominent early feature of DPN and can be severe, despite minimal signs of DPN. Patients describe their feet as “tight,” having painful prickling, burning, electrical, sharp, or jabbing sensations [5].

From this perspective, this review was designed to delineate a general overview of the topic referring to the conventional therapies in use but, first and foremost, possible unconventional, natural and safe treatments. Some studies report how the trend today is increasingly directed to self-care measures, adopting complementary and alternative medicine (CAM), which seem to yield better results in terms of quality of life [6]. CAM practices and self-care encompasses health-protective behaviours, utilisation of preventive medical services, symptom evaluation, various self-treatment practices, and interaction with the informal medical care sector [7].

Emerging evidence show that physicians do not always know much about CAM treatments which are prescribed more for their safety than for their effectiveness [7,8]. No doubt an informational campaign would serve to wider dissemination and could initiate a cultural revolution in the medical field.

For this reason, the most common practises applied in oriental therapies as well as compounds more or less believed active into counteract the development of the pathology will be here reviewed. Many of these include suggestions still on an experimental basis, but that hint at the possibility of drawing from natural sources for the retrieval of active principles that could be forward-looking in the clinical practice.

## 2. Diabetes Mellitus in Numbers

Statistical numbers related to diabetes mellitus are not negligible. According to data updated to March 2013 provided by the World Health Organization, there has been an increased incidence of diabetes cases from 1980, when 153 million patients were recorded, to 2008, in which 347 million people worldwide have diabetes mellitus (DM). Moreover, it is believe that this amount will rise to 380 million in 2025, representing 7.1% of the world’s adult population [9]. This means that diabetes is a major health problem afflicting millions of people across high-, middle- and low income countries [10].

A total of 57 million deaths occurred in the World during 2008, of which 36 million (63%) were due to non-communicable diseases (NCDs, comprising mainly cardiovascular diseases, cancers, diabetes and chronic lung diseases). Among these, diabetes alone caused 1.3 million deaths [11], therefore we can define it as a global health problem. Today, over 90% of diabetic patients are diagnosed with type 2 diabetes mellitus (T2DM) [12], with an increased incidence in recent years probably due to an increased prevalence of its main risk factors (obesity and sedentary life) as well as to a declining mortality likely explained by the improvements in treatment [13]. Moreover, an improved survival of these patients is accompanied by the typical complications of chronic diabetes, the most relevant of which, grouped with retinopathy and nephropathy [14], can be considered the onset of DPN, that affects dramatically from 8.3% for patients with newly diagnosed T2DM to up to 50% in patients who have had diabetes for 25 years [15]. National health systems and patients have to support a huge economic health care burden—in the USA the estimated cost of diabetes just in 2007, was approximately 174 billion of dollars for 17.5 million adults with diabetes [12]. In a report of the Italian agency of the drug (Agenzia Italiana del FArmaco, AIFA) about medications prescribed for diabetes treatment in the period 2002–2011, yearly prescription averages have increased by approximately +1.9%. Noteworthy, an increase of 14.3% in the prescription of the biguanide metformin, a first line treatment in the T2DM management [16] was recorded, confirming the positive spread of its use as a drug of first choice.

## 3. Neuropathic Pain in DPN and Other Pathologies

This section has not been designed to be an exhaustive review about neuropathic pain, but just an illustrative compendium to give the idea how, commonly, pain and in particular, neuropathic pain can link together some diseases with different etiology, in particular neurological/neurodegenerative disease with DPN that born as secondary complication of DM, metabolic disease [17] (Table 1). 

Notably, pain is one of the first typical manifestations of ongoing inflammation [18]. Other possible causes of nociceptive pain include: traumatic events, burns, post-operative pain or disease-related pain. Moreover, in terms of duration, it can be classified as *acute* or *chronic*; under the profile of its localization it can be *local* or *diffuse*; *somatic*, *visceral* or *mixed* whether we consider the anatomical origin of pain stimulus. More specifically, a clear summary of the concept of “pain” can be given as follow: (A) nociceptive pain: a protective sensation associated with the detection of potentially tissue-damaging noxious stimuli; (B) inflammatory pain: a consequence of the tissue damage and of the infiltrating immune cells (it lasts as long as the cause remains); (C) pathological pain: as sanctioned by the International Association for the Study of Pain (IASP) it is a damage to the peripheral or central nervous system (neuropathic) [19,20] or an abnormal function (dysfunctional) [21] due to input and output wrong signals culminating in a uncontrolled and often unconditional sensation of pain [22].

Under normal circumstances, pain is an intense and unpleasant sensation but the capacity to experience this sensation is fundamental to the preservation of bodily integrity [23]. Pain is the reason for 40% of patient visits in a primary care setting, and about 20% of these have had pain for longer than 6 months [24]. Focusing on chronic pain, and in particular, talking about NP, it can broadly to be defined as a condition resulting by a neurological secondary event, following to a main pathology. It can develops in many different ways, timing and severity and the precipitating cause is not always easy to identify [25]. Moreover, it is difficult to treat because of its severity, chronicity and resistance to simple analgesics [26]. NP is a common condition found in many pathologies, characterized by peripheral nerve fibers that, for different reasons (mainly demyelination), transmit incorrect signals to the brain regarding peripheral pain centres, causing neurological dysfunction painful sensations in the absence of actual harm, or affecting the surrounding areas to the actual presence of a disorder, which then become highly sensitive to pain.

**Table 1:** Comparative therapies to treat NP associated to DPN as well as to neurodegenerative diseases.

Disease	Pain	Main symptoms	Therapies
DPN	Frequently, patients with DPN live together with painful sensations such as, allodynia, (pain due to a stimulus that does not normally provoke pain) or hyperalgesia, which is an increased response to a stimulus that is not normally painful [27].	Neuropathic pain paresthesia tingling, or numbness, loss of feeling in the hands, arms, feet and legs.	Gabapentin Pregabalin
MS	MS is a disease related to nervous system lesions, frequently leading to pain symptoms, that in turn, could be responsible of psychological complication [28].	Neuropathic pain as secondary ailment to demyelination, neuroinflammation and axonal damage in the central nervous system. Lhermitte’s sign Trigeminal neuralgia spasticity-related pain [29,30,31].	Sativex^®^ oromucosal spray [31]: 9-Δ-tetrahydrocannabinol/cannabidiol (1:1) Lyrica^®^ Dronabinol [32]: Bedrocan—Cannabis Flos 19%, Bedrobidiol—Cannabis Flos 12% Bediol—Cannabis Flos 6% Peripheral antiepilettic drug [33] (Oxcarbazepin)
GBS	Pain syndromes of GBS has been reported in up to 89% of patients of both neuropathic as well as nociceptive origin. Preclinical data suggest an immune pathogenesis of neuropathic pain.	Paresthesia/neuropathic pain, particularly in the acute phase [34,35].	Corticosteroid [36] Gabapentin [34] Carbamezapine [37]
PD	Pain is a non-motor symptom of Parkinson’s disease: 70%–80% of patients with Parkinson’s disease (PD) suffer from painful sensations mainly neuropathic, followed by nociceptive. There is a higher prevalence of peripheral neuropathy in PD compared with controls, sometimes associated with low vitamin B12 levels, elevated methylmalonic acid levels and cumulative levodopa exposure [38].	The most common clinical types of pain experienced by the patients were: dystonic pain (48%), paresthesia/neuropathic pain (36%) musculoskeletal pain (28%). The PD patients described their physical experience of pain as: aching (46%), a feeling of tension (18%), sharp pain (12%), deep pain (12%), dull pain (11%) [39,40].	Common therapy for neuropathic pain [41]: tricyclic antidepressants, serotonin-noradrenaline reuptake inhibitors(SNRIS) topical lidocaine, calcium channel α–2δ ligands (gabapentin and pregabalin), opioid analgesics [42].

Almost one-third of patients with diabetes mellitus have been diagnosed with NP [43], in particular, polyneuropathy, (or diabetic peripheral neuropathy, DPN), a common and debilitating complication of diabetes, in 16%–26% of diabetic patients characterized by pain in the feet as sharp, stabbing, or burning [44]. Thus, sharing some feature of inflammation, in agreement with Votrubec’s description, NP could be defined as cold or electric shocks and may be associated with tingling, pins and needles, numbness or itching [25]. In particular, it is a direct consequence of a macro- or microscopically identifiable lesion, or an identifiable disease process affecting the somatosensory system [21]. This classification of pain shows clinical signs of spontaneous pain, parasthesia, and mechanical and thermal hyperalgesia or allodynia [45], a sensation of pain in response to normally nonpainful stimuli [44].

Moreover, although DM is classified mainly as a metabolic disorder, NP can be considered a common feature with other NCDs, particularly with neurodegenerative pathologies.

Neurodegenerative disorders such as Multiple Sclerosis (MS), Guillain Barrè Syndrome (GBS) and Parkinson’s Disease (PD), which are increasingly recognized as the major cause of reduced health-related quality of life [46], often lead to pain and, in particular, to NP through the triggering of a neuroinflammatory mechanism that involves the activation of glial cells, constituting 70% of the total cell population in the brain and spinal cord. [47,48]. Sometimes, the onset of neuropathy can be related to adverse effect due to the pharmacological treatment of the neurodegenerative disease. As in the case of PD patients treated with levodopa, it was shown that they have a high prevalence of NP. In fact, levodopa exposure is associated with fasting methylmalonic acid (MMA) level elevations, which have been related with sensorimotor neuropathy [49]. 

## 4. Conventional Treatments of DPN Associated to DM: Gabapentin *vs.* Pregabalin

Monitoring the status of the glycemic levels of diabetes both in patients with Type 1 and Type 2 diabetes is the first step to prevent most of diabetic complications [50], including DPN. Commonly, one of the most important and severe complications of diabetes mellitus is diabetic peripheral neuropathy [51,52], but an adequate treatment of this secondary pathology has not yet been discovered. Generally, to alleviate pain linked to DPN, the commonly known analgesic drugs, such as non-steroidal anti-inflammatory drugs (NSAIDs) and opiates, represent elective treatments. In the management of DPN, tramadol is often recommended as a second-line analgesic. Chemically, tramadol isn’t a natural molecule but rather a semisynthetic opium-derived compound, made up of a racemic mixture of (+) and (−) enantiomers with different affinity for µ and δ opioid receptors and different effects on the re-uptake of serotonin and norepinephrine [53,54].

A streptozotocin (STZ)-induced diabetic model in rats was performed to assess the antinociceptive effects of the combined administration of tramadol and paracetamol [55]. However, its clinical use is not recommended due to the possible occurrence of epileptic seizures and psychiatric disorders also with low doses. Likewise, also a prolonged use of other analgesic—in particular opium-derived—has revealed relevant physical and psychological adverse secondary side effects (e.g., gastrointestinal side effects, osteoporosis, depression, impaired cognition and a generally poor quality of life) [56]. Moreover, often these compounds have proved to be poorly effective for treating DPN, so that the most efficacy therapies have been obtained through the use of drugs born to treat other diseases or complications, such as anti-epileptics [50,56].

Since NP is the most common and severe manifestation of DPN, common therapy is aimed to alleviate this symptom. Among the above cited therapeutic strategies, gabapentin is broadly given in the UK not only to alleviate general NP, but also in easing symptoms associated with DPN [57]. Its patent has expired and a generic version is available. Gabapentin has replaced the use of carbamazepine in the treatment of patients with DPN, since it has demonstrated significant beneficial effects without the bone marrow suppression and osteoporosis, that previous conventional therapy showed [50].

According to the data released in July 2012 by Italian National Observatory on medicines and focused on the period January-December 2011, the prescription of gabapentin has been slowly and progressively substituted by the prescription of pregabalin (brand name: Lyrica^®^, distributed by Pfizer S.r.l.), the pharmacologically active *S*-enantiomer of racemic 3-isobutyl-γ-aminobutyric acid (a GABA analogue with structural similarity and actions similar to gabapentin) [58]. As of May 2005, pregabalin received regulatory approval in 40 countries, including Italy, for its efficacy in the management and treatment of NP associated with diabetic peripheral neuropathy, postherpetic neuralgia, as well as adjunctive therapy for adult patients with partial onset seizures [59,60]. Nevertheless, pregabalin did not demonstrate significant benefits in terms of efficacy or safety compared to gabapentin. 

Today, in Italy, NP accounted for 0.9% territorial spending with €116.6 million. In particular, the daily consumption of pregabalin is around 1.2 for each 1,000 people. Although pregabalin is an anti-epileptic drug, it is used almost exclusively in the treatment of chronic NP with a steady increase in prescriptions that in the last year touched a value of +13.5% and a peak of increased spending of +13.8%, although from clinical trials, a limited clinical efficacy and however not greater than that of less expensive medications, such as gabapentin prescribed in much lower quantities (0.4 DDD/1000 persons per day) appears (Table 2).

**Table 2:** Prospective comparison between gabapentin *vs.* pregabalin prescription.

	Doses (mg/day)	Defined Daily Dose (DDD/1000 People) ^#^	Δ Reduction of Pain Compared to Placebo	% of Increasing Spending Cost
			(Likert Scale: 0–10 Points) *	(Δ 2011–2010) ^#^
Gabapentin	900–3600	0.4	1.1	1.5
Pregabalin	150	1.2	1.5	13.8
	300			
	600			

* Compared efficacy of gabapentin *vs*. pregabalin in Italy—Data from CeVEAS (http://www.ceveas.it); ^#^ Data from AIFA: Osmed Observatory, Report 2011 [61].

From the European Public Assessment Report (EPAR) for Lyrica^®^ we know that the recommended starting dose of Lyrica^®^ is 150 mg per day, divided into two or three doses. After three to seven days, the dose can be increased to 300 mg per day. Doses can be increased up to twice more until the most effective dose is reached. The maximum dose is 600 mg per day. Stopping treatment with Lyrica^®^ should also be done gradually, over at least a week.

## 5. Alternative Medicine in the Management of DPN

Non-pharmacological approaches and non-conventional treatments into management of DPN patients: acupuncture, electrostimulation and Traditional Chinese Medicine (TCM).

Treatment of neuropathic symptoms (such as pain, paresthesia, numbness) is, to date, a serious problem, particularly due to therapies aimed to treat the pathogenesis of DPN as well as to prevent and treat nerve damage [62] but, unfortunately, with severe side effects. A different way to cure patients with diabetic polyneuropathy symptoms consists in alternative and complementary therapies, that, especially in Europe and the Far East, are more and more accepted as mainstream care [62], used alone or in association with standard pharmacological therapies. These modalities often include a variety of interventions such as use of magnet therapy, laser therapy and more than others, acupuncture [63,64]. This therapy is used to relieve pain, improve well being, and treat acute, chronic, and degenerative conditions in children and adults. In Asian medicine, acupuncture needles are inserted at specific points to stimulate, disperse, and regulate the flow of chi, or vital energy, and restore a healthy energy balance. Acupuncture offers clear clinical advantages in the reduction of symptoms related to nervous disorders. To cite only a few examples, it was reported [64] that a series of six acupuncture sessions associated with ongoing nefazodone therapy showed increased benefit in two of the total three patients. Likewise, n = 63 DPN patients were invited to participate to another study and subjected (n = 42) or not (21) to acupuncture, performed according to the rules of Chinese medicine, including diagnostic palpation to identify sensitive spots. At the end of clinical trial, acupuncture treatment improved DPN symptoms, suggesting that it may accelerate the nerve regenerative process [64]. 

Electrostimulation could also be considered an alternative and non-pharmacological approach to management of DPN [65]. Several studies have demonstrated that electrotherapy may also help in chronic painful peripheral neuropathy associated with diabetes [66,67]. Treatment of pain by electricity application treatment dates back to thousands of years BC: In Egypt and, later, in Greece and in ancient Rome, there was the observation that electric shocks generated by fishes could relieve pain [66]. 

Kumar and colleagues showed >50% of leg pain relief in 42 of the 52 patients with DPN undergoing spinal cord stimulation associated with a better quality of life and functional capacity [68]. Already in 1998, Kumar had obtained preliminary evidence, performing a study on 31 patients with symptoms and signs of peripheral neuropathy. Patients were randomly divided in an unstimulated control group (n = 13 patients) and in a group (n = 18 patients) subjected to electrotherapy via transcutaneous electrostimulation. This cohort was treated at home for a total period of 4 weeks with 4 ms pulse, >2 Hz 30 min/daily at the lower extremities [65]. Results demonstrated that in the 83% of electostimulated-patients (15/18) there was a reduction of pain and of the discomfort associated to peripheral neuropathy [65], without any side effects, concluding that further research should be done to investigate whether this kind of treatment could be designed to be given weekly until the patient becomes pain-free, and then once every month [65].

Another important aspect to treat is the role of natural compounds in oriental Chinese medicine as alternative treatments for DPN patients. Over the centuries, the importance of natural products for medicine and health has been enormous [69]. Today the research efforts are aimed to discover new potential drugs that avoid the always more and more frequent side effects, while guaranteeing a safety treatment. From this perspective, oriental—especially Chinese—medicine is a forerunner. In China, herbal medicines to treat DPN are used separately or in combination with conventional medicines [70]. Overall, this natural, alternative and complementary medicine consists of interventions administered orally or topically, classified as self-prescribed herbal formulas, single use of herbs or patented Chinese herbal medicines [70].

In the theory of Chinese medicine, health is a state of balance between yang and yin, between Qi (life energy) and Xue (blood), and between the five elements of wood, fire, earth, metal, and water [71], so that from the nature come all a series of remedies well known as TCM. TCM hypothesizes in four bullet points the possible causes—etiology and pathogenesis—leading to the development of DPN [72]:

1. Yin (black) or yang (white) deficiency. They are part of the eight principles of TCM (the other six are: Cold and heat, internal and external, deficiency and excess) and their unbalance generates an inner cold;
2. Increased duration of disease in diabetes, alterations in the fluidity of the blood (viscosity) with consequent alterations in the blood circulation and afflux to organs and tissues (blood stasis, microvascular coagulation, blockage of sinews and channels);
3. Obesity followed by spleen and stomach impairment;
4. Tissue necrosis as well as muscles, sinew, and channels altered physiology, also due to deficiency of liver and kidney.

In 2005, Feng *et al.* highlighted the beneficial properties of natural products. Traditional medicinal herbs classified as aldose reductase inhibitors seem to prevent and delay diabetic complications, such as diabetic nephropathy, vasculopathy, retinopathy, peripheral neuropathy [73]. In particular, among this class of compounds, great inhibitory capability was ascribed to flavonoids such as quercetin, silymarin, puerarin, baicalim and berberine [73]. 

Moreover, as results from a Medline search of “medicinal herbs and diabetic peripheral neuropathy”, Pubmed lists sixteen articles, one of the most recent of which [74], interestingly, cites a list of herbs with pharmacological properties for DPN, that includes: Astragalus Radix (traditional Chinese name (TCN): Huang Qi), Angelicae Sinensis Radix (TCN: Danggui), Pheretima (TCN: Dilong), Chuanxiong Rhizoma (TCN: Chuanxiong), Codonopsis Radix (TCN: Danshen), Carthami Flos (TCN: honghua), Hirudo (TCN: shuizhi), Rehmanniae Radix (TCN: dihuang), Spatholobi Caulis (TCN: jixueteng), Paeoniae Radix Rubra (TCN: chishao), Cyathulae Radix (TCN: chuanniuxi), Asari Radix and Rhizoma (TCN: xixin), and Scolopendra (TCN: wugong). Pharmacological properties of these medical plants against DPN have been compared with vitamin B12 activity, that, when deficient, particularly in metformin-treated diabetes patients [70,75], has been linked with diabetic neuropathy and functional disabilities [76]. In particular, Astragalus Radix has been demonstrated to possess Qi-tonifying properties [74,77,78]. In general the family of astragaloside herbs, that include for exemple Astragaloside IV (Radix Astragalus membranaceus Bunge), is reveled to be effective on DPN.

Moreover, Chuan Xiong Rhizoma has proven to be a blood circulation-promoting drugs, while both Angelicae Sinensis Radix (Dang Gui) and Paeoniae Radix Rubra (Chi Shao) seem to have blood-tonifying activity [74].

Another compound well known in TCM is guineensine, extracted from *Piper longum*, that seems to possess therapeutic effects against DPN [79].

Finally, also experimental studies corroborate the value of TCM. The most recent research of the year 2013 on Pubmed about “diabetic peripheral neuropathy and alternative medicine” lists six papers, the latest of which shows beneficial effect of Tang-Luo-Ning (TLN) against DPN in streptozotocin-induced diabetic rats [80]. TLN is an effective Chinese recipe in TCM for the treatment of DPN [17,18]. According to the theory of TCM, the TLN recipe mainly contains three different traditional Chinese herbs: Huang Qi, Chishao and Salvia miltiorrhiza Bge. (Danshen). The study highlights the great capability of TLN which offers a promising alternative medicine for the DPN patients to delay the progression of the disease. 

## 6. Phenolic Compounds

Since diabetes has been known as an oxidative stress disorder caused by imbalance between free radical formation and the ability of the body’s natural antioxidants, many studies have suggested that oxidative stress can play a role in systemic inflammation, endothelial dysfunction, impaired secretion of pancreatic β-cells and impaired glucose utilization in peripheral tissues [2], that lead to long-term secondary complications.

The current literature describes some beneficial effects against DPN, derived by the use of natural phenolic compounds. A large amount of vegetables and fruits contain phenolic acids, to which many physiological and pharmacological functions are assigned, since they could inhibit oxidative stress induced by free radicals and protect photooxidation [81]. *In vitro* and *in vivo* experiments have shown that phenolic acids exhibit powerful effects on biological responses by scavenging free radicals and eliciting antioxidant capacity. Additionally, phenolic acids were also found to exhibit anti-inflammatory, antiallergic, antimutation effects, and inhibit cardiovascular diseases. Moreover phenolic acids, such as chlorogenic acid, syringic acid and vanillic acid, have been suggested to possess cytoprotective ability in the prevention of diabetic neuropathy complication via a mechanism that goes against neuronal cell-cycle regulatory failure followed by apoptosis [82]. 

Hydroxytyrosol, a polyphenolic olive-derived compound with several biological activities, in particular anti-oxidant properties demonstrated effects both in humans [83] and experimentally, where it was shown to counteract the secondary NP associated to streptozotocin-induced diabetes in rats [52]. 

To find new alternative compounds to treat DPN is today a goal of primary importance. An ongoing study conducted at Rajiv Gandhi University of Health Sciences is aimed at testing the antioxidant properties against DPN of *Terminalia bellirica* Roxb, that was yet reported to have hypoglycaemic activity in rats [84]. This plant, the major ingredients of which are ellagic and gallic acids, is one of the oldest medicinal herbs of India and an ingredient of Indian Ayurvedic drug triphala, normally used for treatment of digestion and liver disorders [85]. 

More and more examples are given from Nature, where antioxidant compounds seem to be a very good resource against DPN [86]. Methanol extract from *Allium cepa*, the common onion, has shown more significant improvement than *Allium sativum*, well known as garlic, in the treatment of diabetic neuropathy in mice, maybe due to its higher content of phenolic compounds [86]. 

An animal study provides an exemple of the use of *Momordica Charantia Linn.* (Cucurbitaceae) fruit, one of the most famous traditional plants worldwide, also well known as bitter melon [87], tested in an experimental model of streptozotocin-induced diabetic neuropathy in rat. Summarizing the results of the study, in addition to the well-known antihyperglycemic and antioxidant properties associated with neuroprotective activity reported by other authors [87], a new capability to counteract DPN is ascribed to the extract derived from *Momordica Charantia* [88].

## 7. Cannabinoids and Alkaloids

Cannabinoids are a class of diverse chemical compounds that activate the surface cannabinoid receptors, currently classified under two subtypes, termed CB1 and CB2. The ligand-receptor interaction represses neurotransmitter release in the brain. In particular, agonists include the endocannabinoids (naturally produced in humans and animals), synthetic cannabinoids and phytocannabinoids. The most abundant cannabinoids, enclosed in the plant arising to this last group, is the phytocannabinoid ∆9-tetrahydrocannabinol (THC), well known to be the primary psychoactive compound of the *Cannabis sativa.* Cannabidiol (CBD) is another major constituent of the plant, representing up to 40% in its extracts.

Actually, the most famous compound belonging to this class is a pharmaceutical composition developed in Great Britain and that goes under the name of Sativex^®^. From the official GW Pharmaceutical (the company that, to date, holds the drug patent [89]), we can see that this spray preparation, a composition of Δ9-tetrahydrocannabinol/cannabidiol, is approved in the UK, Spain, Germany, Denmark, the Czech Republic, Sweden, New Zealand and Canada to treat mainly spasticity associated to MS [90], cancer pain [91] and NP of various origin, including DPN [92,93].

Probably its use in common clinical practice is limited by its psychotropic component that long-term could cause depression as a side effect [92]. However, patients with psychotic disorders, pregnant or breastfeeding woman should avoid the use of cannabinoids, despite the relatively low acute toxicity. Cannabis side-effects vary and depend on several variables (administrated dose, route of administration and the patient’s state of mind), but in any cases, cannabinoids prolong the times of reaction and decrease concentration. Moreover, after sudden break from long-lasting use, withdrawal symptoms can appear, although they entirely disappear after a week or two [94].

The alkaloid class encloses all natural compounds containing an amine group that confers basic properties to the molecule associated with pharmacologic effects. Among hundreds of compounds, to list just some famous examples of alkaloids, we can cite cocaine (from the leaves of the *Erythroxylum coca* plant), morphine (extracted by “poppy pods”- *Papaver somniferum-*), papaverine (an opium alkaloid antispasmodic drug analogue of morphine), serotonin (a monoamine neurotransmitter found in roots, leaves, fruits and seeds from at least 42 species [95], such as dates, papayas and bananas) as well as caffeine (a bitter, white crystalline xanthine alkaloid found in varying quantities in the seeds, leaves, and fruit of some plants such as coffee and tea bush, as well as from kola nut, yerba maté, guarana berries, guayusa, and the yaupon holly), theobromine (the main alkaloid present in *Theobroma cacao*) and capsaicin ((6E)-N-(4-Hydroxy-3-methoxybenzyl)-8-methyl-6-nonenamide, the active and primary pungent component of chili peppers - *Capsicum annuum* [96]). Capsaicinoids, resiniferanoids, unsaturated dialdehydes and triprenyl phenols represent the four known chemical classes of naturally occurring vanilloids [96]. In particular, some recent papers summarize the beneficial effects of topical application of a cream containing 8% capsaicin dispensed in patch form to treat peripheral NP [97,98]. Topical skin application of the cream causes enhanced sensitivity, followed by a period with reduced sensitivity and, after [92], persistent desensitisation, so that a single patch application of 30–90 min seems provide significant pain relief for up to 12 weeks in some people with chronic pain [97]. Unfortunately, there are unknown risks, especially on epidermal innervation, of repeated application for long periods and the high cost of this composition has slowed its spread.

## 8. Fatty Acids: Pharmacology and Evening Primrose Oil Importance

Linoleic acid is the main dietary essential fatty acid (EFA). In 1950s, in a study performed on diabetic rats by Brenner and colleagues the activity of Δ-6-desaturase, a key enzyme that converts linoleic acid (LA) to γ-linolenic acid (GLA, an essential component of myelin and neuronal cell membrane) was discovered and assessed. This is the first reaction that leads to the preferential formation in turn of prostaglandin E1 (PGE1with anti-inflammatory, antiplatelet, and vasodilating properties) or, according to a slower reaction, to arachidonic acid by-products such as prostaglandin E2 (PGE2), leukotrienes and thromboxane, promoting inflammation, vasoconstriction and platelet aggregation [99,100]. Diabetic animals showed a greatly impaired activity of the enzyme, with the consequence that they require much more linoleic acid than normal animals [101]. Moreover, following this discovery, it was demonstrated that patients with type 1 and type 2 diabetes mellitus display a compromised Δ-6-desaturase activity [99], with decreased levels of PGE1 and increased levels of PGE2 and thromboxane [102]. Dietary supplementation with products rich in GLA, such as evening primrose oil, has been suggested since it increase the production of PGE1 [99].

Evening primrose oil, a rich source of Ω-6 essential fatty acids (primarily GLA and LA), is extracted from the seeds of *Oenothera biennis*. Commercial preparations of evening primrose oil are typically standardized to 8% GLA and 72% LA [99]. Particularly, a recent study on the use as antioxidant of a mixture of α-lipoic acid and evening primrose oil has evidenced an improvement in neuropathic pain through increased PGE1 synthesis [103].

**Table 3:** Summary of alternative approaches and treatment found to counteract DPN.

Kind of Therapy/	Genus/Species
Class of Compounds	Subclass of Compound
Acupuncture	
Electrotherapy	
Oriental Chinese Medicine	Astragalus Radix
	Angelicae Sinensis Radix
	Pheretima
	Chuanxiong Rhizoma
	Codonopsis Radix
	Carthami Flos
	Hirudo
	Rehmanniae Radix
	Spatholobi Caulis
	Paeoniae Radix Rubra
	Cyathulae Radix
	Asari Radix and Rhizoma
	Scolopendra
	Astragaloside IV
	(Radix Astragalus membranaceus Bunge)
	Guineensine, extracted from Piper longum
	Tang-Luo-Ning mainly contains three different traditional Chinese herbs: Huang Qi, Chishao and Salvia miltiorrhiza Bge. (Danshen)
Phenolic Compounds	*Allium cepa* and *Allium sativum*
	*Momordica Charantia Linn*
	Chlorogenic Acid
	Syringic Acid
	Vanillic Acid
	Ellagic and Gallic Acids
	Hydroxytyrosol (*Terminalia bellirica* Roxb)
Cannabinoids	*Cannabis sativa*
Alkaloids	Cocaine (from the leaves of the *Erythroxylum coca* plant)
	Morphine (extracted by “poppy pods”- *Papaver somniferum-*)
	Papaverine
	Serotonin (roots, leaves, fruits and seeds from at least 42 species, such as dates, papayas and bananas)
	Caffeine (seeds, leaves, and fruit of some plants such as coffee and tea bush, as well as from kola nut, yerba maté, guarana berries, guayusa, and the yaupon holly),
	Theobromine (the main alkaloid present in Theobroma cacao)
Vanilloids	Capsaicin (component of chili peppers—*Capsicum annuum*)
Essential Fatty Acids	Evening primrose oil, extracted from the seeds of *Oenothera biennis*

## 9. Conclusions

The scientific literature is rich in examples of experimental studies aimed at treating DPN. The large amount of examples shown in this paper and finally summarized in Table 3, demonstrates on the one hand that a unique treatment designed to counter DPN, has not been found to date, and on the other that in Nature there are different compounds with possible therapeutic action and that these could be a resource for the future treatment of diseases secondary to diabetes mellitus. Definitely, validation of the hypothesized therapeutic properties of natural compounds as well as further studies in clinical practice are needed. Moreover, these authors are of the opinion that alternative approaches, such as acupuncture and electrostimulation, could be successfully associated and integrated in the management of DPN patients.
